# Task specific inter-hemispheric coupling in human subthalamic nuclei

**DOI:** 10.3389/fnhum.2014.00701

**Published:** 2014-09-08

**Authors:** Felix Darvas, Adam O. Hebb

**Affiliations:** ^1^Department of Neurological Surgery, University of WashingtonSeattle, WA, USA; ^2^Colorado Neurological Institute and Electrical and Computer Engineering, University of DenverDenver, CO, USA

**Keywords:** subthalamic nucleus, Parkinson’s disease, deep brain stimulation, local field potentials, oscillations, phase synchronization

## Abstract

Cortical networks and quantitative measures of connectivity are integral to the study of brain function. Despite lack of direct connections between left and right subthalamic nuclei (STN), there are apparent physiological connections. During clinical examination of patients with Parkinson’s disease (PD), this connectivity is exploited to enhance signs of PD, yet our understanding of this connectivity is limited. We hypothesized that movement leads to synchronization of neural oscillations in bilateral STN, and we implemented phase coherence, a measure of phase-locking between cortical sites in a narrow frequency band, to demonstrate this synchronization. We analyzed task specific phase synchronization and causality between left and right STN local field potentials (LFPs) recorded from both hemispheres simultaneously during a cued movement task in four subjects with PD who underwent Deep Brain Stimulation (DBS) surgery. We used a data driven approach to determine inter-hemispheric channel pairs and frequencies with a task specific increase in phase locking.We found significant phase locking between hemispheres in alpha frequency (8–12 Hz) in all subjects concurrent with movement of either hand. In all subjects, phase synchronization increased over baseline upon or prior to hand movement onset and lasted until the motion ceased. Left and right hand movement showed similar patterns. Granger causality (GC) at the phase-locking frequencies between synchronized electrodes revealed a unidirectional causality from right to left STN regardless of which side was moved.Phase synchronization across hemispheres between basal ganglia supports existence of a bilateral network having lateralized regions of specialization for motor processing. Our results suggest this bilateral network is activated by a unilateral motor program. Understanding phase synchronization in natural brain functions is critical to development of future DBS systems that augment goal directed behavioral function.

## Introduction

Neuronal assemblies are rhythmically activated and inhibited through the influence of an oscillatory extracellular local field potentials (LFPs). This phenomenon has been demonstrated in neocortex (Murthy and Fetz, 1996; Womelsdorf et al., 2007; Fröhlich and McCormick, 2010) and subcortical structures (Moran et al., 2012). Thus, the recorded LFP, thought to represent the average synaptic input to the dendritic tree, influences individual neuronal firing not only by the underlying dendritic input to individual neurons, but on the population level via oscillations in the extracellular medium. A macroscopic example of this synchronization phenomenon can be seen in the London Millennium pedestrian bridge video footage on its opening weekend. Random pedestrian footfalls induced a resonant frequency in the bridge, and led to further synchronization of footfalls as pedestrians adjusted their steps to stabilize themselves on the oscillating bridge (Dallard et al., 2001).

As the phase of the LFP influences the probability of neuronal firing, phase locking of specific frequencies in distant regions of the brain implies connectivity between these regions for the transfer of information (Fell and Axmacher, 2011). The study of cortical networks and particularly quantitative measures of their connectivity has been of considerable interest in the study of brain function (Singer, 1993; Buzsáki and Draguhn, 2004; Palva et al., 2005). There exists now a wide variety of methods to quantify functional connectivity, based on signal coherence (Gross et al., 2001), phase coupling (Lachaux et al., 1999), Granger causality (GC; Blinowska et al., 2004) and various non-linear measures (Canolty et al., 2006; Darvas et al., 2009a; Greenblatt et al., 2012). Similar to signal coherence, phase coherence may be used to measure direct synchronization between two cortical sites in a narrow band. Synchronization of the phase of the alpha (8–12 Hz) or beta (13–30 Hz) bands can be observed in ongoing brain activity or in relation to a task with well-defined onset (Simões et al., 2003; Palva et al., 2005; Bar et al., 2006). While the concept of phase synchrony is straightforward, its practical application to brain activity without a priori knowledge of anatomical connections is challenging. For example, non-trivial cross-talk effects across channels, particularly in non-invasive recordings such as electroencephalography (EEG) or magnetoencephalography (MEG), must be accounted for or eliminated. For both non-invasive and invasive recordings, there are many potential interaction sites (~n^2^, where *n* is number of recording sites) that can generate spurious results. Therefore data driven applications of phase synchrony have a high computational demand, and require applied statistical approaches that capture only genuine synchrony across distinct brain regions.

Phase analysis of depth-electrode recordings permits a simplified data driven approach, because these recordings are obtained from very few sensors implanted in cortical or subcortical structures. Recordings from left and right sub-thalamic nucleus (STN) in patients suffering from Parkinson’s disease (PD) during awake-surgery present a unique opportunity to test for functional synchronization. Despite the lack of direct anatomical connections between left and right STN, there is evidence for physiological connectivity or synchrony (Novak et al., 2009; Walker et al., 2011; Hebb et al., 2012). Typical clinical examination procedures in PD exploit this physiological connectivity to enhance the motor signs of PD (Powell et al., 2011). Our understanding of this physiological connectivity of STN between hemispheres is limited. Studies of ipsi-lateral recordings (Levy et al., 2000, 2002; Brown et al., 2001; Williams et al., 2003), demonstrate modulation of synchronization within one hemisphere for ongoing and induced activity. We have previously reported bilaterally symmetric STN power modulation of beta frequency oscillations during language and motor tasks (Hebb et al., 2012). In consideration of this finding, we hypothesized that a movement task leads to synchronization of neural oscillations in bilateral STN. We implemented phase coherence, a measure of phase-locking between two cortical sites in a narrow frequency band, to demonstrate this synchronization.

Here, we present our findings of phase synchrony and causal interaction in STN between hemispheres in relation to a specific task, i.e., the movement of the left or right finger in four patients suffering from PD.

## Materials and methods

### Subjects

Four subjects undergoing Deep Brain Stimulation (DBS) as standard of care for treatment of idiopathic PD were enrolled in this study. All subjects provided informed consent for participation in this research study, in a manner approved by the internal review board of the University of Washington. Details of the DBS surgery have previously been published in Hebb et al. (2012).

### Paradigm/Task

The motor task in this study consisted of cued button presses using the thumb of each hand. Each task block consisted of 15 presses and was performed with the same hand, alternating right and left sides for successive blocks. Speech and speech/motor combination tasks were also performed but not analyzed for this study. A detailed description of the entire paradigm has been previously published (Hebb et al., 2012). In addition to button press feedback, forearm electromyography (EMG) was recorded synchronously with LFP recordings from the STN. The cue for each action was indicated acoustically and inter-cue interval duration was randomized to minimize anticipatory effects.

### Signal recording analysis

#### Recording

**Figure 1:**
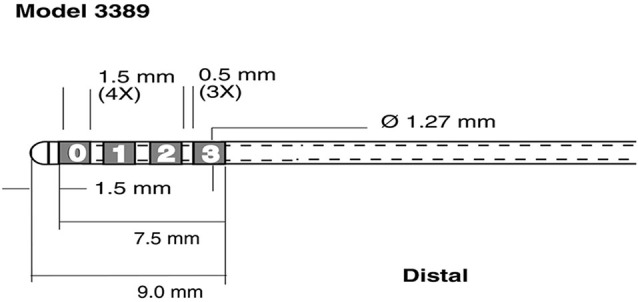
**Schematic representation of recording electrodes used for Local Field Potential (LFP) recordings**. Medtronic 3389 DBS lead (reprinted with the permission of Medtronic, Inc. © 2008). The four leads were rearranged into 3 bipolar pairs C1 = 1-0, C2 = 2-1 C3 = 3-2 for each hemisphere.

LFP and EMG were digitized at 5 kHz, using a SynAMP2 (Neuroscan, Victoria, Australia) biosignal amplifier. LFP recordings were referenced to a linked-mastoid ear reference. For each STN (i.e., in the left and right hemisphere) data was recorded from the four contacts of the DBS lead (Medtronic 3389, Minneapolis, MN, see Figure 1).

#### Preprocessing and segmentation

The four raw LFP signals per hemisphere were referenced to their neighbors (channel 1—channel 0 = bipolar C1, channel 2—channel 1 = bipolar C2, and channel 3—channel 2 = bipolar C3, see Figure 1) to yield 3 bipolar signals. All data was down sampled to 500 Hz prior to further analysis to reduce computational burden. The data was segmented into epochs based on EMG recorded for each arm. We band pass filtered the continuous EMG signal between 70 and 90 Hz and applied a Hilbert transform to the narrow band filtered signal, to compute the time varying analytical amplitude of the EMG. We used a 95 percentile threshold of the analytic amplitude, taken over the whole recording, to detect movement onset. There were between 59 and 90 trials per subject and between 30 and 45 left hand movements. Data was segmented from 2 s prior to 2 s post EMG onset. To avoid edge artifacts in our signal analysis, we considered a period from 1.5 s prior to EMG onset to 1.5 s post EMG onset. The mean delay for each subject between cue and onset of EMG activity was between 435 ms and 272 ms. Across all subjects, 95% of all delays across trials were smaller than 800 ms; hence we defined the baseline from 1.5 s to 0.8 s prior to EMG onset. We did not extend the baseline before 1.5 s to avoid overlap with prior trials.

#### Signal analysis

For each of the four subject’s datasets, there were three right and three left hemispheric channels (C1R/L, C2R/L, C3R/L) in each STN. While the STN was the target of the DBS electrode, the total physical electrode span is greater than the anatomical span of the STN. For the purpose of this analysis, the placement with respect to specific STN regions was considered arbitrary and hence a direct comparison of electrode pairs across subjects was not possible. Therefore, to determine task specific interactions we considered all pairs in our analysis. Given the low number of available subjects (four) and the spatial incongruence between montages across subjects, analysis aimed to identify significant common task related features in each subject.

We used the phase locking value (PLV; Lachaux et al., 1999) to test for movement related synchronization of rhythms across hemispheres. The PLV, unlike coherence, can capture transient changes in synchronization, e.g., due to task onset, and measures the degree to which two signals align their relative phase at a given frequency with respect to some external event. This measure has been successfully applied in the neurosciences to describe long range cortical interactions (see Rodriguez et al., 1999; Simões et al., 2003; Bar et al., 2006).

There were 9 possible symmetric pair interactions across hemispheres and 18 possible asymmetric interactions. Symmetric pairs are (C1R, C1L), (C2R, C2L), and (C3R, C3L), where the order of the hemispheres is irrelevant in calculating the PLV measure, e.g., PLV(C1R, C1L) = PLV(C1L, C1R). Such symmetry does not exist for GC analysis.

We computed the PLV using a complex Morlet wavelet transform to determine the time-varying phase and amplitude for each STN inter-hemispheric LFP pair and for each trial. This method has been shown to be equivalent to phase extraction use the Hilbert transform on band-pass filtered signals (Le Van Quyen et al., 2001), but has the additional advantage of providing optimal time-frequency resolution. We then computed the PLV from the wavelet phase. As the PLV measure is symmetric with respect to its input signals, i.e., PLV(x,y) = PLV(y,x), any lateralization effects that might occur by changing from right to left thumb will not be detected by this measure. Therefore, the right and left button press trials were combined to compute the average phase difference across all trials.

Initially we computed the PLV over a wide range of frequencies, from 6 to 40 Hz in 1 Hz steps after band-pass filtering the signals from 3 to 50 Hz. We converted this map to Z-scores (Bar et al., 2006), based on the pre-movement baseline interval from −1.5 s to −0.8 s to identify significant changes in synchronization due to movement. To test our hypothesis of a change in synchronization from baseline, we integrated the Z-scores over a time interval from −0.1 to 1 s, which covered the whole movement period, as measured by the EMG. This integration resulted in one value per frequency bin and allowed us to identify frequencies of interest. By screening for relevant frequencies first, we avoided having to run time consuming calculations over a wide range of frequencies in our subsequent analysis.

Since PLV values in the baseline interval were not normally distributed, the Z-scores and their integrals over an interval were interpreted as a measure of relative change in synchronization and were not considered a test for significance. In order to identify significant changes, we subjected our frequency dependent mean Z-scores to non-parametric permutation tests. While parametric descriptions of the PLV exist (Darvas et al., 2009b; Aydore et al., 2013), we used a non-parametric test, which is free of assumptions about baseline synchronization of the STN and was appropriate for values derived from the PLV, such as the integrated Z-scores. In order to distinguish genuine synchronization from (a) induced changes in power; and (b) volume conduction effects, we applied two permutation schemes successively. For (a) we computed the PLV after shuffling the trial order of one of the two channels (Lachaux et al., 1999), since the PLV is sensitive to a particular trial order, while power changes are not. For (b) to avoid zero-phase lag synchronization, e.g., due to volume conduction, we computed the phase lag index (PLI; Stam et al., 2007) for each channel pair and frequency deemed significant by test (a). The PLI tests for symmetry of the distribution of instantaneous phase differences around 0 or Pi. A PLI of zero indicates a symmetric distribution or instantaneous lag and a PLI of −1 or 1 indicates a negative or positive non-zero phase lag. Assuming an equal probability under the null hypothesis of a zero phase-lag, the null hypothesis distribution of the PLI is given by the binomial distribution with the number of trials as parameter. For each subject we had 35 frequency bins and 9 channel pairs, resulting in a maximum of 315 independent tests. However we expected the actual number of independent tests to be lower since neighboring frequency bins are correlated and different channel pairs can contain a common member, e.g., (C1R,C3L) and (C1R,C2L). At the most conservative Bonferroni correction for *p* < = 0.05/315, we need a minimum of 6300 permutation tests to determine any significant frequencies. We ran 63,000 trial shuffling tests per subject, to provide a sufficient resolution of the empirical determined *p*-values.

Finally, to determine the direction of the interaction, we computed the Granger Causality (GC), using the CCA Matlab toolbox (Seth, 2010), between the significant pairs and frequencies. GC has been established as a measure of directed, causal interaction for neurophysiological signals (Kamiński et al., 2001; Hesse et al., 2003) and is based on a linear autoregressive model of two or more time series. In this model, a signal *X* is assumed to be causal to *Y*, if the modeling error for a linear model of *Y* based on its own past is greater than the modeling error based on a linear model that involves both signals *X* and *Y*. Causal interaction of specific rhythms can be derived from the Fourier representation of these models.

Like the PLV, GC is sensitive to trial order, so significance can be established based on a trial shuffling permutation scheme. We computed GC over short time windows to determine the time course of causality.

## Results

**Figure 2:**
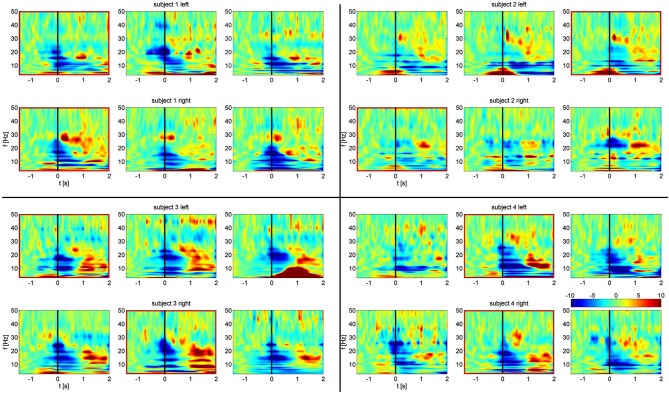
**Frequency-time spectrograms of relative power changes in each STN bipolar channel for all four subjects arranged from left to right: C1, C2, C3.** Top rows: left STN. Bottom rows: right STN. Spectrograms are normalized per frequency based on a −1.5 s to −0.8 s baseline period. Channels outlined in red show significant task related alpha frequency phase synchronization. Movement onset defined by EMG is at time zero, indicated by the black line.

All subjects exhibited movement dependent suppression in the alpha and beta band followed by a beta rebound at the end of the movement, a finding described previously (Hirschmann et al., 2011; Hebb et al., 2012; Litvak et al., 2012). Time frequency maps, normalized to a baseline pre-movement period are shown in Figure 2. While multiple channel pairs exhibited changes in power, we found synchronization in all four subjects in a single channel pair at low alpha frequencies within a very narrow band (7, 8, 8, and 9 Hz). There were no significant changes in beta synchronization, despite the presence of behavioral modulation of beta power. Furthermore, the same analysis, carried out for segmentation based on cue, rather than EMG onset, showed no significant synchronization.

**Figure 3:**
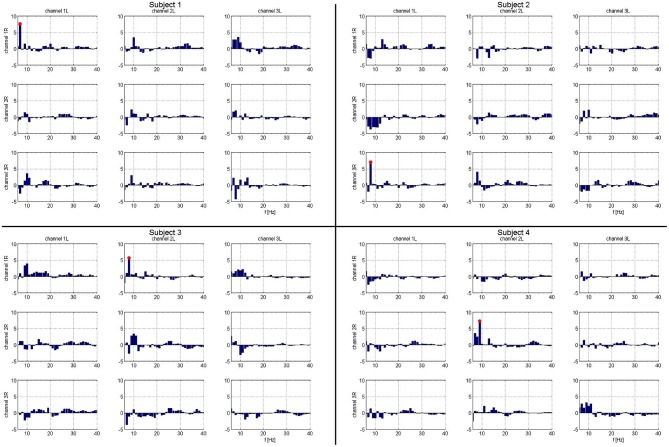
**Matrix of frequency specific phase locking analysis for each subject.** Column headings are left hemispheric channels: 1L, 2L, 3L. Row headings are right hemispheric channels: 1R, 2R, 3R. Frequencies that show significant task related phase locking for each left-right STN channel pair and subject. Frequencies marked in red are significant at a corrected *p* < 0.05. For each subject, there is exactly one combination of electrodes with significant phase locking.

A summary of the integrated PLV value over time is shown in Figure 3. Synchronization was concurrent with finger movement, but did not depend on which side was moved. While relative alpha power was low during the task, this does not imply absolute low alpha amplitude, which would impact the accuracy of the phase. Due to the paradigm design, the relative decrease in alpha power during movement can be attributed to changes from an increase in alpha power prior to movement (Klostermann et al., 2007).

**Figure 4:**
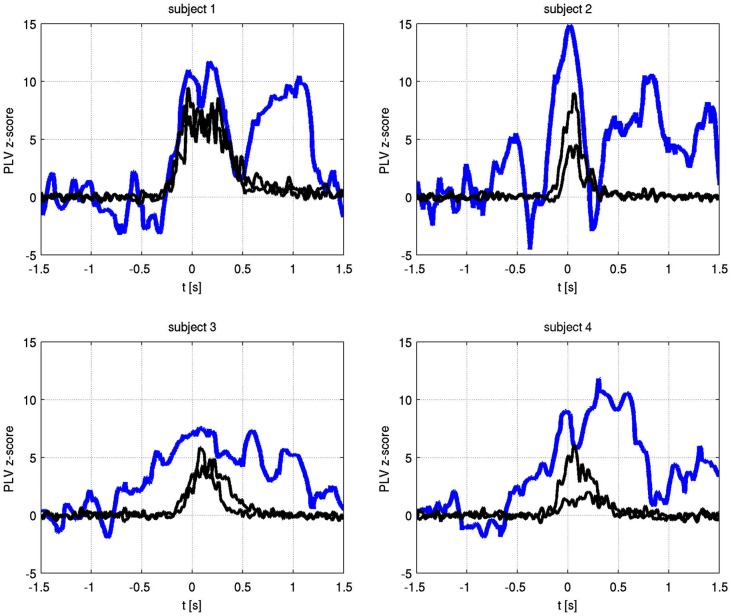
**Time varying alpha frequency phase locking value (blue) and EMG activity for both hands (black) for each subject**. The PLV has been transformed to Z-scores, using −1.5 s to −0.8 s as the baseline period. Only the significant channel pair for each subject is shown (subject 1: C1 right to C1 left, subject 2: C3 right to C1 left, subject 3: C1 right to C2 left, subject 4: C2 right to C2 left).

The temporal evolution of the alpha phase locking of identified frequencies is shown in Figure 4. In all subjects, phase synchronization increased over baseline at or prior to movement onset and lasted for the duration of finger movement.

**Figure 5:**
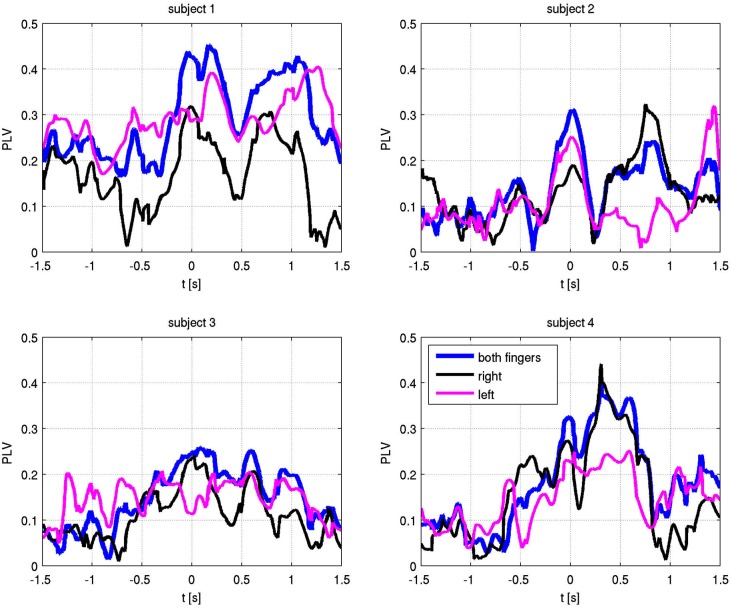
**Phase locking values (PLV) with respect to EMG-defined motor task onset at time zero**. Alpha frequency absolute PLV for both fingers (blue), right finger only (black) and left finger only (magenta).

**Figure 6:**
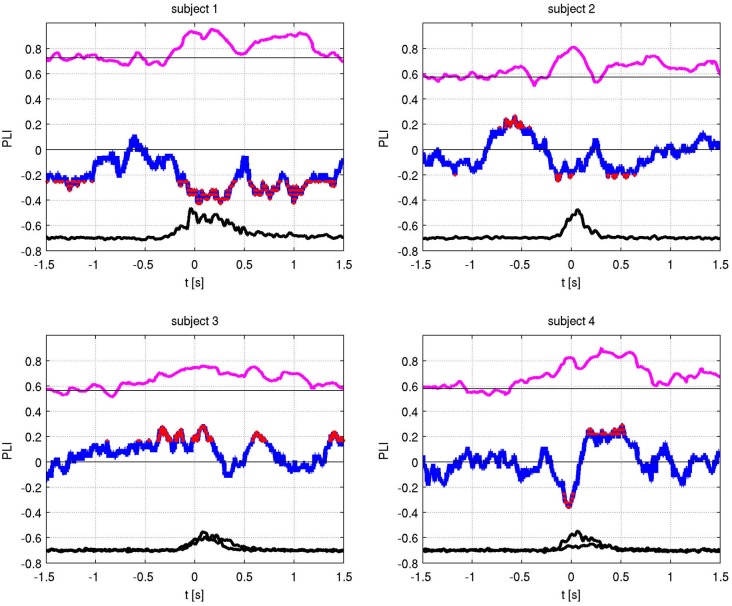
**Phase locking value (PLV) (magenta) and Phase lag index (PLI) (blue) with EMG activity (black)**. The PLI measures the consistency of the phase-locking across trials. Red dots indicate times of significant (i.e., non-zero) phase lag at *p* < 0.05 (corrected).

The absolute values of the PLV for both fingers and individual sides are shown in Figure 5. In all patients, left and right movement showed a similar activation pattern. We also show the PLI, with PLV values for both fingers and EMG in Figure 6. Values significantly above or below zero indicate lag or lead of either side (left or right STN). We marked significant values of the PLI with red dots based on a non-parametric permuation test. While there was no consistent lag/lead pattern across subjects, all exhibited stable non-zero phaselag at movement onset and throughout movement. Since the PLI does not quantify the relative phase-lag, this value cannot be used to infer absolute lag times. However it is useful to asses the stability of the PLV and to confirm that the observed effects were not due to volume conduction effects (i.e., zero PLI). Also, a stable phase lead or lag as observed in the alpha synchronization here indicated that low amplitudes in the alpha band, e.g., due to the relative drop in alpha power during movement, do not affect our overall phase-synchrony estimation.

**Figure 7:**
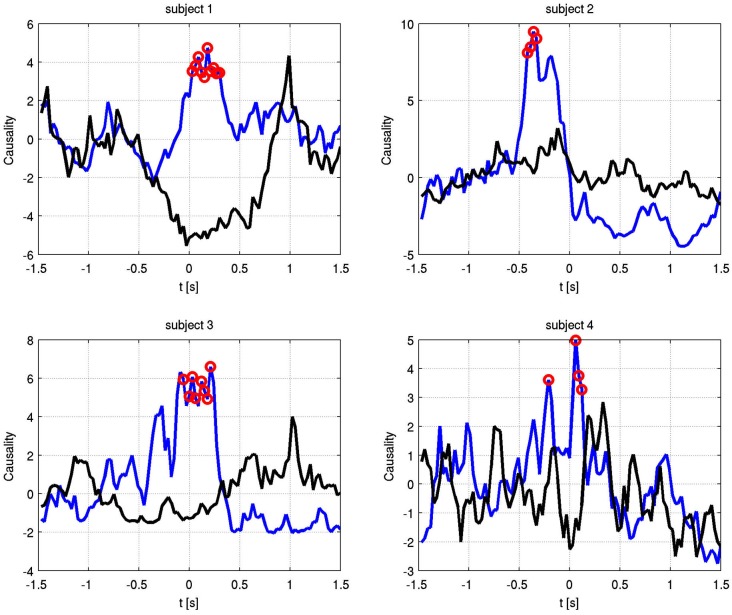
**Granger causality between phase-locked channel pairs at the significant alpha frequencies for each subject over time**. Blue shows right to left causality, black vice versa. Red dots indicate significant increase in causal flow. In all four subjects, there is unidirectional right to left flow, regardless of the finger that is moved.

**Figure 8:**
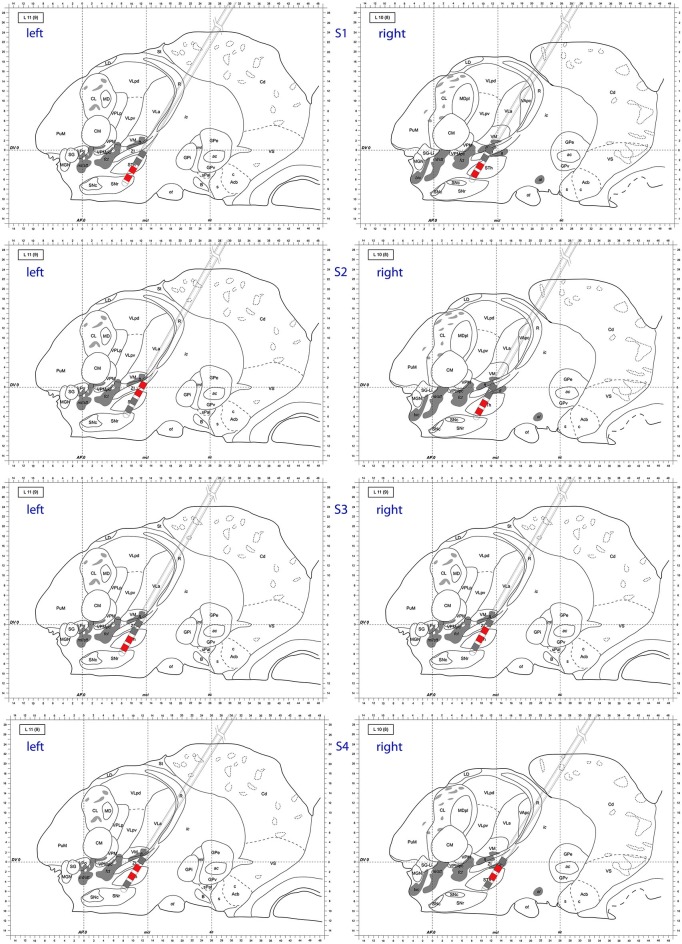
**Atlas representation demonstrating DBS lead position for each subject**. Bipolar montages with cross hemispheric phase synchrony are colored in red. The left column corresponds to left hemisphere, right column to right hemisphere. Sagittal atlas adopted from Morel (2007) with permission.

Also at these frequencies, we observed task dependent increases in the causality from the right STN to the left STN, independent of which side was actually moved (Figure 7). This flow was unidirectional from right to left and peaked after EMG onset. When this causality was significant, according to our permutation test, we marked these times with red dots. We also showed the causality measure in the opposite direction. The interacting electrode pairs are shown in Figure 8, where the bipolar montages for each channel, highlighted in red, are superimposed on an atlas of the subcortical structures.

## Discussion

### Unilateral motor task activates bilateral subcortical motor network

Phase synchronization across hemispheres between basal ganglia structures implies the existence of a bilaterally connected network for motor processing. Our results suggest that a simple unilateral motor program activates this bilateral network. Alpha oscillations recorded from bilateral STN LFP in particular were phase synchronized across hemispheres at the onset of a button press task. These significant findings are the first demonstration of alpha oscillatory synchronization at movement onset between STN. Also, this study found that Granger causal flow of synchronization was always directed from the right to left STN, regardless of which hand performed the motor task. If the GC was an artifact of the ongoing phase synchronization between the two signals, one would expect equally high causality for both directions. However we find no significant increase in causality for the opposite direction, i.e., interaction from left STN to right STN.

These findings support that basal ganglia functionality may not simply be mirrored within each hemisphere to provide processing for each motor cortex. Rather, lateralized regions of this bilaterally distributed basal ganglia network may drive particular sub-specialized functions. Lateralized basal ganglia functionality has previously been ascribed to functions such as language, with the dominant hemisphere basal ganglia structures supporting cortical language function (Hebb and Ojemann, 2013). However, the concept that there may be lateralized specialization of the basal ganglia for motor program initiation, a function that both motor cortices perform equally and independently, is novel.

Our study has important limitations. Due to the invasive nature of these recordings, this study did not include normal control subjects. Our findings could be specific to PD. The involved alpha frequencies are in the range of the pathologic oscillatory network observed in PD, and electrical stimulation at these frequencies is associated with worsening of motor symptoms (Timmermann et al., 2004). Our study was limited to four subjects due to limited surgical research opportunity in subjects undergoing bilateral simultaneous DBS lead implantation. Therefore, no meaningful group level analysis could be performed.

However, due to the universal and individually significant findings across all subjects, using robust data driven methods with conservative non-parametric statistical methods, we feel that these results represent a generalizable phenomenon, although this should be explored in a larger sample size.

### What is the significance of phase synchronization?

Synchronization of distant neuronal assemblies may arise from the influence of a third party, such as the thalamus, a well-connected nucleus capable of driving phase synchrony in multiple brain regions. Alternatively, low frequency rhythms recorded in neuronal field potentials may represent a resting state that must be overcome to allow meaningful computation. For instance, beta oscillatory power in motor cortex is most prominent at rest and immediately after a motor action (beta rebound). This resting state oscillation may serve to end motor programs and suppress involuntary movements, and acute damage to key regions may lead to undesired movements (hemiballism, STN stroke). Phase synchrony is likely to have multiple roles, and these roles may be dependent on frequency and the distance spanned.

### What is the generator of the alpha rhythm?

Rhythmic oscillations of the LFP may to arise from a closed loop network system between member nuclei with inherent delays in their connections that define natural resonant frequencies (Holgado et al., 2010; Jaeger and Kita, 2011). Two potential candidate generators for LFP rhythms are the thalamo-cortical loop and the Globus Pallidus pars externus–STN loop. The alpha band phase synchronization that we observed with movement onset was recorded across hemispheres, but was not global or widespread. Phase synchronization was quite specific to certain bipolar pairs of DBS electrodes, indicating that even within the small volume of the DBS electrode span, this phase synchronization is localized. As the basal ganglia contains parallel pathways through its members, recording from a cross section of a basal ganglia nucleus (e.g., STN) may allow for monitoring of multiple independent short or long range oscillating channels.

The origin of basal ganglia oscillations is unclear, and the origin may vary with the frequency of the oscillation. One theory is that LFP oscillations are locally generated in the basal ganglia. This is supported by computational models and slice preparations of basal ganglia (Plenz and Kital, 1999; Holgado et al., 2010). It is possible that time limited synchrony between hemispheres arises from a synchronizing effect of a stochastic external influencer (i.e., prefrontal cortex). An alternate hypothesis is that oscillations are a result of sustained long range communication between the cerebral cortex and the basal ganglia. In this sense, both the oscillations and the synchrony of the oscillations between hemispheres represent sustained long range information flow and processing rather than a stochastic effect from an influencer external to the oscillation generator. These two hypotheses are subtly different. The first hypothesis recognizes the basal ganglia as a group of nuclei that are autonomously creating a type of neural oscillation or neural noise that serve to modify, select, and refine input from external sources. The latter more classic view considers the basal ganglia as a group of nuclei standing by to process external information and is kept busy by the constant barrage of information flowing from the cortex into the system. This classic view of the basal ganglia has not been able to explain the various deficits in PD (Olanow et al., 2009).

### Role of basal ganglia oscillations in function and disease

If the basal ganglia operate as an autonomous system, serving to filter, select and refine competing information from the cerebral cortex, the phenomena of stochastic resonance (SR) applied to cortical input signals may provide a basis for describing this functionality. Briefly, SR counter-intuitively increases the signal to noise ratio of neural information by adding an optimal level of noise (Moss et al., 2004). Chakravarthy (2013) suggested that the basal ganglia may facilitate willed actions using SR. High levels of synchronized neural activity in the STN and Globus Pallidus pars internus are neural correlates of PD, and so the underlying pathophysiology of PD may impair the basal ganglia’s ability to apply SR in facilitating movement. This also helps to explain why low frequency DBS pulse trains may worsen the motor signs of PD, and low amplitude random DBS pulses may have superior efficacy compared with traditional high frequency DBS pulse trains (Tass et al., 2012). In relation to the current study, the transient alpha phase synchronization we observe may be the result of a transient cortical signal that is amplified by the SR mechanism.

In addition to the synchronization, we observed a causal relationship between hemispheres that was independent of which side was moved. The directional aspect of the synchronization could have been a general phenomenon related to movement or attention related to the task. Our behavioral paradigm may have activated a bottom-up signaling pathway initiated by the audio cue, leading to bilateral alpha phase synchronization. Regions of the prefrontal cortex are known to be well connected to the striatum and directly to the STN (Aron et al., 2007). Strong external cues for experimental paradigms are linked to activity of the right inferior frontal gyrus, potentially accounting for the right hemisphere leading in motor program initiation (Aron, 2011). It would be straightforward to conclude that the right to left GC was solely a result of the bottom-up external cue processing through right inferior frontal gyrus to right STN and potentially a parallel pathway across the corpus callosum to regions of the left frontal cortex to left STN. However, when our analysis was conducted on the basis of the cue timing rather than EMG movement onset, the causality measures were not significant. Therefore, we conclude that our causality findings arise from motor program initiation rather than sensory processing of the audio cue to perform the action.

We attempted to identify anatomic sub-regions within the STN where the phase synchronization was detected to look for patterns (ventral to ventral, dorsal to dorsal, etc.). However, as depicted in Figure 8, we were not able to determine a generalized pattern across subjects. This is potentially due to anatomical variations of parallel neural pathways through the STN, or to inherent measurement limitations within the small volume of the STN. However, as we analyzed signals from differential electrode montages which are sensitive to local potential changes only, the observed synchronization is genuinely restricted to STN sub-regions and is not due to some global effect.

### Review of the STN node in neuronal assembly phase synchronization

Phase synchronization between bilateral STN has been previously reported. Cross hemispheric STN Beta frequency coherence in the off medication state was reported by de Solages et al. with a peak coupling frequency of 27 Hz (de Solages et al., 2010). Little et al. reported that beta frequency range PLV between STN is reduced with the administration of levodopa, but they did not report an effect on alpha range synchronization nor did they describe movement induced changes in phase locking (Little et al., 2013). The time delay for cross hemispheric STN-STN synchrony has been measured in PD patients during rest in the medication OFF state to correspond to the period of theta, beta, and gamma frequency bands. This supports the theory of multiple oscillatory channels through the basal ganglia that likely serve different anatomical pathways and functions (Silchenko et al., 2010). Thus, our current work adds to this literature by identifying another frequency band, alpha, that synchronizes across hemispheres at the initiation of a motor task.

Given no direct anatomical connection between left and right STN, the current findings imply the existence of a closed STN-cortex-STN loop or parallel connections from cortex to bilateral STN through which synchronization occurs across hemispheres (Carpenter and Strominger, 1967). This could potentially occur via corpus callosum. Since our study did not include recording of cortical activity with EEG or MEG, we can only speculate on the global pathways of this synchronization. In support, previous studies (Crowell et al., 2012; de Hemptinne et al., 2013; Shimamoto et al., 2013) provide evidence for connections through the sensorimotor system on the cortical side.

Several studies have shown movement-dependent frontal cortex to STN synchronization within a range of specific frequency bands. These studies used computational techniques that combine both the phase and amplitude of oscillations. In combined MEG and STN LFP recordings, resting baseline has been characterized by beta frequency STN-cortex coherence with M1 to STN directionality, whereas motor activity was characterized by gamma frequency STN to M1 directional coherence (Litvak et al., 2012). Another MEG-LFP study, analyzing resting state data, observed beta coherence between STN and ipsilateral sensorimotor and premotor regions, and alpha coherence between STN and temporal regions (Hirschmann et al., 2011). This group later reported that the beta range coherence was reduced by movement, but the superior temporal gyrus-STN alpha band coherence was modulated neither by movement nor by medication (Hirschmann et al., 2013). Using a directed transfer function to calculate bidirectional information flow in EEG and STN-LFP data sets, a different study found beta frequency coherence was greater between cortex and STN at rest than during movement, and gamma coherence was greater in the medication on state compared with the off state (Lalo et al., 2008). In another study utilizing combined EEG and STN-LFP data sets, there was a decrease in the beta frequency range event-related coherence measures during observation of movement and actual performance (Alegre et al., 2010). Beta frequency oscillations recorded in the basal ganglia also influence (couple with) high frequency gamma oscillations in the frontal cortex. In studies of combined STN LFP and motor cortex electrocorticography, the amplitude of the gamma frequency power was modulated by the phase of the beta frequency rhythm, a relationship termed cross frequency coupling (Canolty et al., 2006; de Hemptinne et al., 2013). While considerable attention had been paid to beta band modulation in these reviewed studies, Klostermann et al. (2007) observed STN alpha band changes in a similar paradigm congruent with our findings. With respect to movement, these authors report an increase of STN alpha power prior to onset and a subsequent drop with action. The alpha (8–9 Hz) coherence between the cortex (EEG) and STN decreased at the cue and recovered with action. Since our data analysis is timed to movement onset, the movement related increase in PLV shown in our study corresponds to the recovery of coherence reported in Klostermann et al. (2007). In summary, we have presented evidence for oscillations recorded in the STN to synchronize with, and modulate, rhythms in the cerebral cortex. This relationship allows for cross hemispheric influence via cortical-cortical connectivity.

## Conclusions

Phase synchronization between bilateral basal ganglia structures supports the existence of an inter-hemispheric network with lateralized regions specialized for motor processing. Our results suggest that a simple unilateral motor program activates this bilateral network. Understanding phase synchronization in natural brain functions will be critical for the development of the next generation of DBS systems that minimize the negative impact on, and potentially augment, goal directed behaviors.

## Conflict of interest statement

The authors declare that the research was conducted in the absence of any commercial or financial relationships that could be construed as a potential conflict of interest.

## Abbreviations

PD: Parkinson’s Disease
STN: Subthalamic Nucleus
LFP: Local Field Potential
EEG: Electroencephalography
MEG: Magnetoencephalography
EMG: Electromyography
PLV: Phase Locking Value
PLI: Phase Lag Index
SR: Stochastic Resonance
GC: Granger Causality.
